# Construction and use of a prokaryotic expression system for *Helicobacter pylori* AhpC

**DOI:** 10.1186/1756-0500-5-328

**Published:** 2012-06-25

**Authors:** Khalid Mehmood, Fariha Hasan

**Affiliations:** 1 Department of Microbiology, Quaid-i-Azam University, Islamabad, 45320, Pakistan; 2 Microbiology and Environmental Toxicology Department, University of California Santa Cruz, Santa Cruz, CA, 95064, USA; 3 Department of Pharmacy, Hazara University, Havelian, Abbottabad, Pakistan

**Keywords:** Prokaryotic expression system, *H. pylori*, *tsaA*, Alkyl hydroperoxide reductase, Recombinant fusion protein, AhpC

## Abstract

**Background:**

*Helicobacter pylori* is an important pathogen responsible for human gastric problems like inflammation, ulcers and cancer. It is widely prevalent in developing countries with low socioeconomic status. Since the infection remains asymptomatic in most individuals, efforts for efficient diagnostic markers to identify high risk patients are warranted. In this study, we constructed an expression vector that overexpresses the *H. pylori* AhpC protein as a glutathione S-transferase fusion protein. We furthermore examined whether this recombinant fusion protein retained immunogenicity and thus would be useful as a diagnostic marker.

**Findings:**

The full-length *tsa*A gene from *H. pylori* strain G27, which encodes AhpC, was cloned in plasmid vector pGEX-6P-2 to create the recombinant plasmid vector *pGEX-tsaA*. The nucleotide sequence of the clone showed 100% homology with corresponding published sequence of original gene. Over-expression of the target protein GST-AhpC was achieved in *E. coli* BL21 (DE3) cells by induction with isopropyl-beta-D-thiogalactoside (IPTG). GST-AhpC was extracted and identified using SDS-PAGE as a 52 kDa protein. Western blotting results using commercial antibodies against whole cell *H. pylori* showed that the fusion protein retained immunogenecity.

**Conclusion:**

A recombinant prokaryotic expression system was successfully established with high expression efficiency for target fusion gene *pGEX-tsaA*. The expressed GST-AhpC protein showed immunoreactivity against commercial anti-*H. pylori* antibodies. This recombinant fusion protein can be developed as a diagnostic marker for screening patients with chronic *H. pylori* infections.

## Findings

### Background

*Helicobacter pylori* has been recognized as a human gastric pathogen able to colonize in the stomachs of around half of the world’s population [1]. Most infected individuals remain asymptomatic, however, the infection may cause acute and chronic gastritis or peptic ulceration, besides being a risk factor for development of gastric adenocarcinoma, mucosa-associated lymphoid tissue (MALT) lymphoma and primary gastric non-Hodgkin’s lymphoma [2-5].

*H. pylori* infection is acquired in early childhood. Like all developing countries, the prevalence of *H. pylori* infection in Pakistan is very high in children. Results of urea breath test in infants from suburbs of Karachi revealed that 80% were positive for *H. pylori*[6]. *H. pylori* is able to colonize human stomach for life, if not eradicated. Persistent colonization requires *H. pylori* to avoid damage from by-products of oxygen metabolism and oxidative host responses. *H. pylori* has an impressive array of antioxidant proteins. The bacterium protects itself against such oxidative damage by expressing enzymes like superoxide dismutase SodB [7], catalase KatA [8] and KatA-associated protein KapA [9]. The activities of alkyl hydroperoxide reductase AhpC [10], thiol peroxidases Bcp and Tpx [11] have also been reported to protect *H. pylori* against organic peroxides. NADPH quinone reductase MdaB [12] and the iron-binding protein NapA [13] were also found involved in resistance to oxygen stress.

*H. pylori* AhpC is a thioredoxin (Trx)-dependent AhpC and a member of the 2-Cys peroxiredoxin family (2-Cys Prxs). AhpC is one of the major proteins for antioxidant defense in *H. pylori* and plays an important role in gastric colonization by the microbe [10]. The *tsaA* gene was originally annotated as HP1563 in *H. pylori* 26695 [14] and HP1471 in *H. pylori* J99 [15], however Chalker *et al.* (2001) annotated the gene as *ahpC*, because this nomenclature is more usual for bacterial alkyl hydroperoxide reductase enzymes [16]. AhpC has been reported in many bacteria, like *Escherichia coli* and *Salmonella typhimurium*[17]. *H. pylori* AhpC was reported much near to eukaryotic Prxs unlike reductases found in many other bacterial species and indeed, could act like a molecular chaperone similar to Prxs present in yeast and human [18]**.** Recently, AhpC was found to be consistently expressed in higher amounts in *H. pylori* strains isolated from gastric cancer patients than in patients presenting gastritis only [19]. The 26-kDa protein first reported as an antigenically conserved *H. pylori* species specific protein, is now being predicted to be a useful diagnostic marker for detection of *H. pylori* infection [20]. It was also found associated with a specific antibody response in patients with adenocarcinoma [21].

In the present study, a recombinant expression plasmid containing whole *tsaA* gene from *H. pylori* G27 was constructed. The plasmid was cloned in *E. coli* BL21 cells and recombinant fusion protein was expressed, extracted, identified and analyzed for immunoreactivity with commercial anti *H. pylori* antibodies. The results of this initial work provide a basis for future studies using this fusion protein to develop a specific diagnostic marker for detection of advanced stage diseases like peptic ulcer, gastric cancer and adenocarcinoma due to chronic *H. pylori* infection in Pakistani population.

### Results

#### Polymerase chain reaction, cloning and transformation

**Figure 1:**
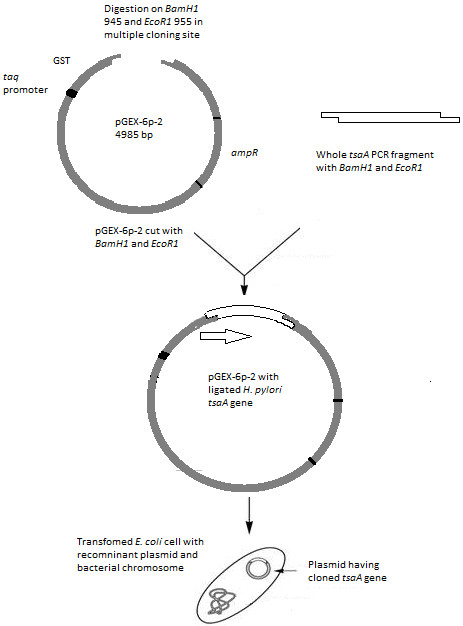
**Schematic diagram of cloning and transformation of *tsaA* gene.** Whole *tsaA* gene having *BamH1* and *EcoR1* sites was ligated with *pGEX-6p-2* that had been cut with the same enzymes to generate *pGEX-tsaA.* This plasmid was used to transform *E. coli*, which produces rGST-AhpC fusion protein under induction with IPTG.

**Figure 2:**
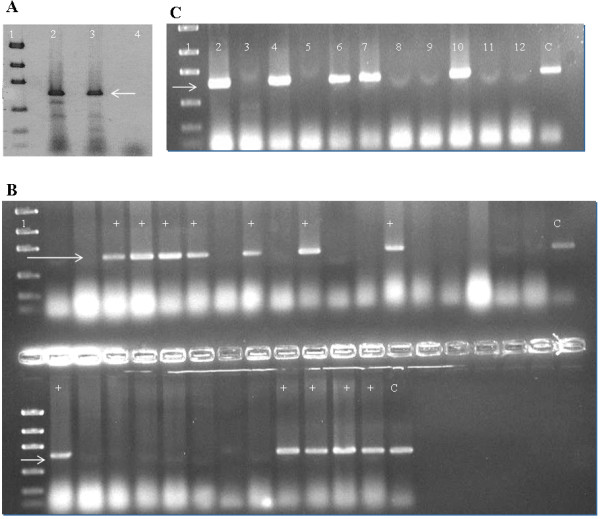
**A. Target amplification fragments of *tsaA* gene.** Lane 1: Low mass ladder (New England Biolab); Lanes 2 & 3: Target amplification fragments of *tsaA* gene (~600 bp) form G27 and positive control 26695; Lane 4: Blank control. **B**. PCR showing *E. coli* BL21 colonies after cloning, positive for *tsaA* gene by direct colony PCR method. Lane 1 is low mass ladder. Lanes with + sign show successful transformants. **C**. PCR for *tsaA* using plasmid extracts of transformed *E. coli* BL21 colonies. Lane 1 is low mass ladder. Lanes with bands at target site (~600 bp) show successful transformants.

The full-length *tsaA* gene was amplified as described in the methods, digested with restriction enzymes and ligated with *pGEX-6p-2* that had been cut with the same enzymes to generate *pGEX-tsaA.* This plasmid places the GST coding sequences N-terminal to the TsaA/AhpC coding sequences and thus should generate rGST-AhpC fusion protein (Figure 1). *pGEX-tsaA* was used to transform *E. coli* DH10B to ampicillin resistance, and the correct nature of *pGEX-tsaA* was verified using PCR and DNA sequencing (Figure 2).

#### Expression of target fusion protein

**Figure 3:**
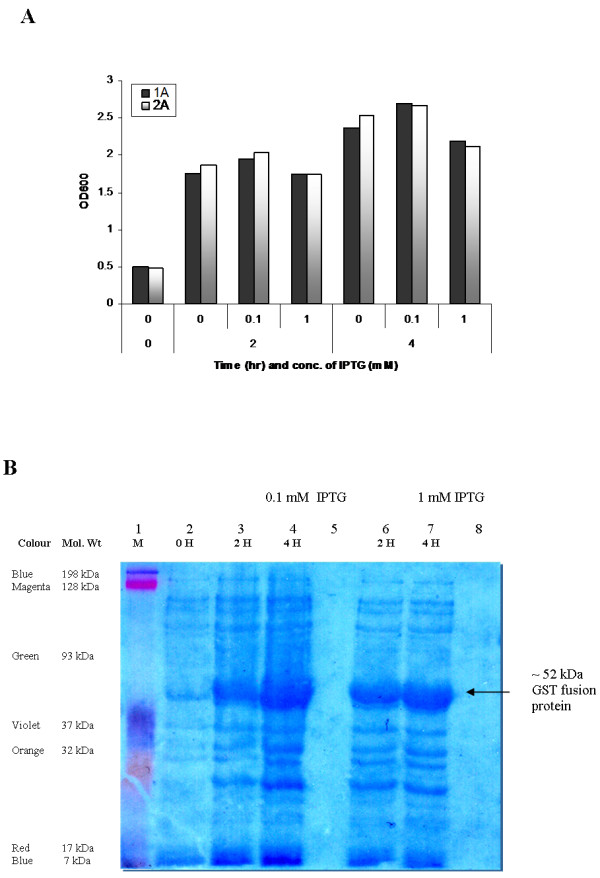
**A. Growth curves showing OD**_**600**_**of *E. coli* BL21 cells for induction of AhpC with 0.1 and 1 mM IPTG at 0, 2 and 4 hours of growth.** 1A and 2A are duplicate samples. **B**. SDS-PAGE showing ~52 kDa target recombinant fusion protein (rGST-AhpC). Lane 1: Kaleidoscope colour marker (BioRad); Lanes 2–4 Protein samples induced with 0.1 mM IPTG at 0, 2 and 4 hours respectively; Lanes 6–7: Protein samples induced with 1 mM IPTG; Lanes 5 & 8: Blank controls.

We next examined whether *pGEX-tsaA* would overexpress rGST-AhpC. For this experiment we transformed *pGEX-tsaA* into the *E. coli* strain BL21 (DE3) that encodes a chromosomal T7 RNA Polymerase under the control of a *tac* promoter. Under IPTG induction, the *tac* promoter gets activated that drives expression of the rGST-AhpC. We thus added IPTG at concentrations of 0.1 and 1 mmol/L to mid-log phase cultures of *pGEX-tsa-* BL21 (DE3) grown at 37 °C, and collected whole cell proteins for SDS-PAGE gel analysis. We found that *pGEX-tsaA* expressed robust amounts of rGST-AhpC that migrated at the expected size of 52 kDa (Figure 3). Further analysis demonstrated that this rGST-AhpC was mainly present as inclusion bodies, based on the observation that the protein was not soluble after cell lysis (Data not shown).

#### Immunoreactivity of GST-AhpC

**Figure 4:**
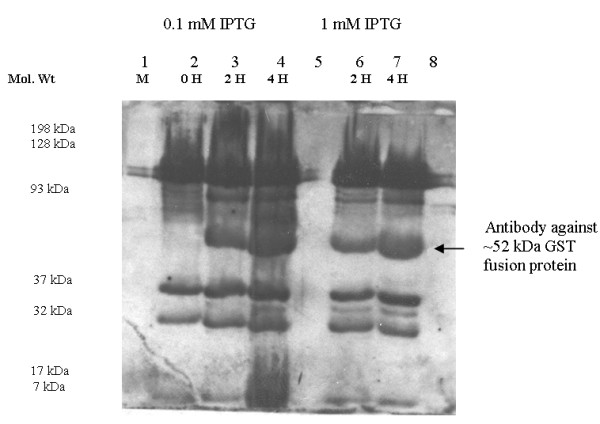
**Western Blot showing detection of target ~52 kDa GST fusion protein by Rabbit anti *H. pylori* antibodies as primary antibody.** Lane 1: Kaleidoscope colour marker; Lanes 2–4: Protein samples induced in *E. coli* BL21 cells with 0.1 mM IPTG at 0, 2 and 4 hours respectively; Lanes 6–7: Protein samples induced with 1 mM IPTG; Lanes 5 & 8: Blank controls.

Although it is well known that antibodies against AhpC are generated during *H. pylori* infection, we did not know if such antiserum would recognize a rGST-AhpC fusion protein. We thus tested whether a commercial rabbit antibody that had been generated against whole cell *H. pylori* would recognize our recombinant rGST-AhpC. We obtained robust recognition, confirming that this recombinant protein still displays critical antigenic determinants (Figure 4).

#### Purification of fusion protein

**Figure 5:**
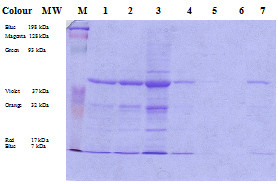
**SDS-PAGE showing different purification steps for fusion protein from pellet after sonication step III (P-III).** Lane M: Kaleidoscope colour marker; Lane 1: Dialyzed pellet before lyophilization; Lane 2: Pellet-III after lyophilization; Lane 3: Pellet-III before lyophilization: Lane 4: Dialyzed pellet after lyophilization; Lane 5: Dialyzed supernatant before lyophilization; Lane 6: Filtrate after concentration and Lane 7: Concentrated fusion protein.

Target fusion protein rGST-AhpC was extracted from large volume LB-Amp culture (1 L) using repeat sonication and washing followed by centrifugation after each step. The samples were checked on 10-12% SDS-PAGE gel for protein quality. Pellet after third sonication and centrifugation step (Pellet-III) showed best results with a good intense band and little degradation/cleavage products. Pellet-III sample was dialyzed and the supernatant, after dialysis was concentrated to approx. 3 ml. Right sized band for fusion protein was observed in case of both dialyzed and concentrated protein samples with no apparent degradation product as compared to pellet-III sample without dialysis and concentration (Figure 5).

### Discussion

The study of virulence factors and important antigens of *H. pylori* is important to understand bacterial mechanisms involved in persistent colonization and inflammation. Prokaryotic expression systems for several important *H. pylori* virulence factors have been successfully constructed and recombinant fusion proteins or their purified proteins used in seroprevalence studies or screened as *H. pylori* vaccine candidates [22-24], however, little attention has been paid towards protein products of housekeeping genes like *tsaA (ahpC)*.

Wang *et al.* (2005) reported that mutant *H. pylori* strains defective in AhpC were more sensitive to oxidative stress conditions compared to wild-type cells [25]. Also, Olczak *et al.,* (2003) reported that *ahpC*/*tsaA* mutant strains were unable to colonize mouse stomach [26]. An important feature of AhpC is that it seems to promote the activity of other proteins. Specifically, loss of AhpC resulted in partial inactivation of catalase, suggesting that AhpC is required for proper catalase activity [27].

At least two studies reported that antibodies against AhpC were found only in *H. pylori* infections that resulted in disease. Wang *et al.,* 2004 found antibodies against AhpC in sera of peptic ulcer, cancer and adenocarcinoma patients infected with *H. pylori*[21]*.* In a recent report, Haung *et al.,* 2011 examined protein expression levels of AhpC in *H. pylori* from different clinical manifestations and observed 5-fold increase in AhpC protein expression in strains isolated from gastric cancer patients than those from gastritis [19]. This describes the potential of AhpC as an efficient biomarker to identify and risk patient population presenting different stages of dseases associated with chronic *H. pylori* infections.

Another important *H. pylori* antigen associated with disease is CagA. Patients infected with *cagA*^+^*H. pylori* have higher risk of developing gastric cancers than those infected with *cagA*^*-*^ strains [28,29]. Various studies have reported that 60 to 70% of *H. pylori* strains isolated from European and North American populations carried the *cagA* gene [30-32], whereas over 90% of the isolates from Asia-Pacific populations were found to be *cagA*^*+*^[33-35]. The fact that nearly all strains of *H. pylori* from countries like Taiwan and China as well as a high proportion in western countries are *cagA* positive makes it difficult to use *cagA* only as a marker for screening high-risk patients in these populations.

The *tsaA* gene from *H pylori* strain G27, cloned in this study, showed 100% nucleotide homology with published corresponding sequences. In the present study, SDS-PAGE demonstrated that the constructed expression systems *pGEX-6p-2tsaA*-BL21(DE3) efficiently produced the target recombinant GST fusion protein, however mainly presented in the form of inclusion body. However, we successfully obtained free of inclusion body fusion protein using repeat sonication and centrifugation protocol which can be extended to subsequent refolding and cleavage from GST tag. The commercial rabbit antiserum against the whole cell of *H. pylori* recognized and combined with rGST-AhpC as confirmed by Western blot, indicating that the fusion protein had high immunoreactivity. However, we could not explain presence of lower intensity bands other than that of the right sized band, both in SDS-PAGE (Figure 3B) and their subsequent recognition in Western Blot (Figure 4). Perhaps these are *E. coli* BL21 (DE3) proteins which cross-reacted with the commercial rabbit antiserum against the whole cell of *H. pylori*. Several other approaches have been used for construction and use of prokaryotic expression system for potential *H. pylori* vaccine candidates like *flaA, flab, ureB* and *cagA* by other groups [22-24].

Limitations of present study include use of GST fusion protein itself and not its cleaved purified product, and not testing the sera of potential patient groups to demonstrate the effectiveness of this approach due to some constraints.

## Conclusions

We have successfully constructed an efficient expression system for *ahpC,* an important gene of *H. pylori* in *pGEX-6p-2*. This construct was successfully transformed in *E. coli* BL21 (DE3) that gave a recombinant fusion protein (rGST-AhpC), mainly as inclusion body. Immunoreactivity against rGST-AhpC was tested against commercial *anti-H. pylori* antibodies raised in rabbit serum. The fusion protein was then processed to get free from inclusion body. This recombinant fusion protein can serve as a potential diagnostic marker for screening advanced clinical manifestations due to chronic *H. pylori* infections. Such strategies are need of the time to identify high risk patients keeping in view the genetic diversity of *H. pylori* regarding a well accepted biomarker CagA across the globe.

## Methods

### Bacterial strains

Bacterial strains used are given in Table 1. *E. coli* strain DH10B was used for cloning and BL21 (DE3) used for protein expression.

**Table 1:** Strains and plasmid used in the study

**Strain**	** *Genotype/description* **	**Source**
** *E. coli* **		
DH10B	Cloning strain	Promega
BL21 (DE3)	*E. coli* B F– dcm ompT hsdS (rB–mB–) gal λ(DE3) [pLysS Camr]	Promega
** *H. pylori* **		
G27	Wild type	Nina R. Salama, University of Washington, Seattle, WA
26695	Wild type	Karen M. Ottemann, UC Santa Cruz, CA
** *Plasmids* **		
*pGEX-6p-2*	GST fusion plasmid	Novagen

### Growth media and chemicals

For solid-medium culture, *H. pylori* was grown on Columbia blood agar (Becton Dickinson) plates with 5% defibrinated horse blood (Hemostat Labs, Davis, CA), 5 μg of trimethoprim/ml, 8 μg of amphotericin B/ml, 10 μg of vancomycin/ml, 50 μg of cycloheximide/ml, 5 μg of cefsulodin/ml, 2.5 U of polymyxin B/ml, 0.2% (wt/vol) β-cyclodextrin (Sigma) (CHBA) at 37°C under conditions of 10% O2, 10% CO_2_ and 80% N2. All antibiotics were from Sigma or ISC Bioexpress. *E. coli* strains were grown on LB Agar or broth with Ampicillin and incubated on 37°C for overnight (LB-Amp). For long-term storage of *H. pylori* strains, 3–5 day growth of *H. pylori* was transferred to brucella broth having 10% FBS (BB10), 1% β-cyclodextrin, 5% dimethyl sulfoxide and 25% glycerol. The cells were frozen at −70°C after pipetting and vortexing.

### DNA extraction

Genomic DNA from 2–3 days culture of *H. pylori* strain G27 was extracted using Wizard genomic kit (Promega) according to manufacturer’s instructions.

### Primers

Primers were designed from genome sequence of *H. pylori* 26695 (HP1563) to amplify the full-length sequence of HP1563/*tsaA* using the U.C. Santa Cruz archeal genome browser (http://archaea.ucsc.edu). Forward primer 5′-CTCTGGATCCATGTTAGTTACAAAACTTGCC-3′ having restriction endonuclease site of *Bam*H1 and reverse primer 5′-CTCTGAATTCTTAAAGCTTAATGGAATTTTC-3′ with *Eco*R1 site were used to amplify full length *tsaA* gene from *H. pylori* G27 genomic DNA. Primers were synthesized by Eurofins MWG Operon, (Huntsville, USA).

### Polymerase chain reaction

PCR reaction mixture contained 100 ng of *H. pylori* G27 genomic DNA, 2.5 μl of dNTPs, 2.5 μl of 30 mM MgCl_2_ PCR buffer, 200 ng of each primer and 1 μl of Taq polymerase**.** Final volume was made to 25 μl with sterile water. Genomic DNA of *H. pylori* 26695 in same concentration was used as positive control. PCR conditions were: Initial melting at 94°C for 3 minutes, then 30 cycles of repeat melting at 94°C for 40 sec, annealing at 52°C for 40 seconds and initial extension at 72°C for 30 seconds, then final extension at 72°C for 3 minutes and storage at 4°C until run on the gel. The results of PCR were observed under UV light after electrophoresis on 1.5% agarose gel stained with ethidium bromide. The expected size of *tsaA* amplification fragment was ~600 bp.

### Sequencing

Sequencing of PCR products was done by the DNA sequencing facility, UC Berkeley, CA. A sample containing 100 ng gel purified DNA and 0.8 pg of forward or reverse primer was adjusted to a total volume of ~13 μl in dH_2_O. Sequences obtained were subjected to nucleotide BLAST feature of National Center for Biotechnology Information (NCBI) website (http://blast.ncbi.nlm.nih.gov/Blast.cgi) for homology analyses with published *tsaA* sequence of G27.

### Cloning of tsaA gene into plasmid vector

Gel purified *tsaA* whole gene was cloned into plasmid vector *pGEX-6p-2.* Both the *tsaA* PCR product and plasmid were digested with restriction endonucleases *Bam*HI and *Eco*RI (New England Biolabs) at 37°C overnight. Digested DNA and plasmid were gel purified and concentration was determined by Nanodrop. The plasmid sample was treated with Shrimp alkaline phosphatase (New England BioLabs). Ligation was done with 0.5 μl T4 ligase enzyme (New England Biolabs) by incubating the ligation mix at 16°C for overnight. This plasmid was transformed into *E. coli* DH10B using electroporation; ampicillin-resistant colonies were screened using direct colony PCR using the original tsaA primers. One colony containing the correct plasmid was used to purify plasmid for sequencing.

### Expression and identification of target recombinant protein

pGEX-Recombinant plasmid was transformed through electroporation into *E. coli* BL21 (DE3) (Promega). One BL21 (DE3) colony of successful transformant containing *pGEX-tsaA* was cultured in LB-Amp broth at 37°C until OD_600_ of 0.5, after which IPTG was added at concentrations of 0.1 and 1.0 and mmol/L. Cultures were incubated for the times indicated in Figure 3A. Cells were then collected by centrifugation at 6000 X g force. The bacterial pellet was resuspended in 200–300 μl of 2x Laemmli sample buffer (1.52 g Tris-Base, 2 g SDS, 20 ml 100% glycerol, 2 ml β-merceptoethanol and 1 mg bromophenol blue in dH_2_O to make 100 ml), and re-suspended pellets were boiled for 5 minutes on water bath for denaturing proteins. SDS-PAGE (10%) was used to measure the molecular mass and output of the target recombinant fusion protein (rGST-AhpC).

### Immunoreactivity of recombinant fusion protein

Immunoreactivity of the fusion protein was then determined. Briefly, PVDF membrane (BioRad) cut to the size of the gel was pre-wet in 100% Methanol. Gel, sponge pads, 4 pieces of Whatman filter paper and MeOH-soaked membrane were soaked in the transfer buffer (1X SDS-PAGE Running Buffer, 20% MeOH) for 10 minutes. The cassettes were closed and placed in the module and contents of the gel transferred to membrane at 250 mA for 75 minutes. The commercial rabbit antiserum against whole-cell *H pylori* (Santa Cruz Biotech. USA) in 1:1000 dilution and HRP-labeling sheep antiserum against rabbit IgG in 1:7500 dilution (Santa Cruz Biotech, USA) were used as the first and second antibodies to determine the immunoreactivity of rGST-AhpC by Western blotting, respectively. Results were visualized using luminol and Biomax Light Film (Kodak).

### Isolation of fusion protein from inclusion bodies

The fusion protein was isolated from inclusion bodies by using an online protocol (http://www.its.caltech.edu/~bjorker/Protocols) with some modifications. Briefly, culture from single colony of successful transformant grown in 1 L LB-Amp broth was centrifuged (Sorvall RC5C Plus, Thermo Fischer Scientific, Rockford, USA) at 7000 X *g* for 20 minutes at 4°C. Resultant supernatant was carefully decanted and pellet was resuspended in 13 ml of solution buffer (50 mM Tris–HCl, 25% sucrose, 1 mM NaEDTA, 0.1% Sodium azide and 10 mM DTT) on ice and sonicated (Sonic Dismembrator, Fischer Scientific, Rockford, USA) at 50% amplitude for 4–5 times on ice. Then 100 μl Lysozyme, 250 μl DNase I and 50 μl MgCl_2_ were mixed with 12.5 ml of lysis buffer (50 mM Tris- HCl, 1% Triton X-100, 1% Na deoxycholate, 100 mM NaCl, 0.1% Sodium azide and 10 mM DTT) and sample was incubated for 45 minutes at room temperature. In next step, 350 μl of NaEDTA was added to the sample and freezed in liquid nitrogen. The sample was then thawed at 37°C for 30 minutes. 200 μl MgCl_2_ solution was added to the solution and placed at room temperature for an hour. Then 350 ml NaEDTA was added again to the solution and placed on ice. This solution was centrifuged at 11000 X *g* at 4°C for 20 minutes and supernatant was discarded in a tube. The pellet was washed with 10 ml washing buffer (50 mM Tris–HCl, 0.5% Triton X-100, 100 mM NaCl, 1 mM NaEDTA, 0.1% Sodium azide and 1 mM DTT) and sonicated on ice. Solution was spun again at 11000 X *g* for 20 minutes at 4°C and supernatant was discarded in a tube. Pellet was again resuspended in 10 ml washing buffer but this time without Triton X-100 and sonicated. The same procedure was repeated for third time. Samples from both supernatant and pellet after each centrifugation step were retained for checking protein quality. In the last step, pellet was dissolved in 5 ml of 8 M Guanidinium solution (pH 8.0) in the presence of 4 mM DTT and shaken at room temperature till complete dissolution. The dissolved sample was stored at −80°C.

### Dialysis and concentration

Solution was diluted to 50 ml with minimal protein buffer (150 mM NaCl and 50 mM Tris–HCl at pH 8.0) and dialyzed against 1 L of MPB using SnakeSkin dialysis tubing (Thermo Fischer Scientific, Rockford, USA) with gentle shaking at 4 °C. After 2 hours of shaking, the buffer was changed and then dialyzed overnight. Solution was pelleted at 3000 X g for 20 minutes at 4 °C. The resultant supernatant was concentrated to about 3 ml volume using Amicon Ultra-15 concentrator (Millipore, USA) following manufacturer’s instructions. Both concentrated and dialyzed pellets were analyzed for proteins on SDS-PAGE. Both the pellet and concentrate were lyophilized (FreeZone 2.5 Freeze Dry Systems, Labconco, Kansas, USA) for long term storage and further use.

## Competing interests

The authors declare no competing interests.

## Authors’ contributions

KM conceived the study, carried out the experimental work and prepared the initial draft of the manuscript. FH helped in result interpretation and preparation of final draft. All authors read and approved the final manuscript.
